# Biomorphic Clothing Sculpture Interface as an Emotional Communication Space

**DOI:** 10.3389/fpsyg.2020.00117

**Published:** 2020-02-07

**Authors:** Young Sun Yoo, Minjin Cho, Jung Sun Eum, Seon Ju Kam

**Affiliations:** Department of Clothing and Textiles, Kyung Hee University, Seoul, South Korea

**Keywords:** biomorphic design, clothing sculpture, interface, parametric design, biophilia

## Abstract

This study found that clothing, combined with digital technology, can be a wearable computer that adapts to environmental change and increases biological limits, as well as an emotional space to which art and design are applied, and that promotes mutual communication. A user-friendly interface is proposed for those pursuing a new artistic experience by building a conceptual algorithm for biomorphic clothing sculpture modeling that can be used to create generative designs simply by entering moderated mediator variables. Biomorphic clothing sculpture refers to clothing that is inspired by life in nature and modeled in three dimensions. As the human body and buildings are 3D forms, the parametric design method used frequently in architecture is implemented here and used as a tool to explore attributes and representations of 3D modeling in clothing sculpture design. On the basis of a literature review, this study presents knowledge-based data for biomorphic clothing sculpture that can predict generative design outcomes, and through a case study, identifies the mediator variables and attributes of the parametric process related to biomorphic clothing sculpture modeling. The knowledge-based data on biomorphic clothing sculpture and biomorphic clothing sculpture modeling mediator variables discovered during the research are applied to the 3D modeling process as visual data and presented as the conceptual interface of biomorphic clothing sculpture.

## Introduction

The recent development of digital technology that crosses the boundary of virtuality and reality has facilitated the transformation and expansion of the concept of space, blurring the lines not only of architecture but also of modeling and formative arts. Moreover, computing technology previously used by designers or architects with expertise is now provided as a service in the form of algorithms on platforms built for design purposes, enabling users to obtain emotional pleasure by creating new sculptures simply by entering moderated data. The parametric design method can be used to create forms that apply structural order and principles that appear in nature based on mathematical algorithms and can automatically modify the features of model components merely by adjusting form-generation figures based on the user’s intuition. Architecture and fashion share the attribute of being a cultural and individual tool of esthetic expression, as both create a 3D form that protects humans. Clothing is defined as a space that covers the human body and, with the mobility of digital humans, is interpreted as an architectural space. Combined with digital technology, clothing can become a wearable computer that adapts to environmental change and increases biological limits as well as an emotional space that is applied to art and design and promotes mutual communication between the artist and the audience.

The biophilia hypothesis suggests that humans feel comfortable in nature from the perspective of evolutionary biology and has become the basic concept of biophilic design (Söderlund and Newman, 2017). Many studies have shown that biophilic design helps humans recover their health and relieves mental stress (Ulrich, 1984; Joye, 2011; Söderlund and Newman, 2017), engaging them and improving creativity (Öhman and Mineka, 2001). Joye and Van Locke (2007) conducted an experiment on stress relief provided by nature and found that simplified natural landscape images and geometrically transformed natural patterns are more effective at reducing stress than real natural environments such as forests. Findings include that elements of biomorphic architecture such as plants, or environments that include plants, are visually appealing and can induce a positive esthetic response (Joye, 2006). Thus, visual elements inspired by living matter in nature stimulate the natural esthetic senses of humans and encourage creativity to produce other artistic objects. Moreover, the advancement of an evolutionary system of computer technology based on a nature-friendly design paradigm promotes design methods based on computing technology that can easily create new geometric forms rooted in nature’s biological structure and elaborate, mysterious biological functions. The objective of this study is to explore parameters required by biomorphic clothing sculpture (BCS) modeling conceptual algorithm to create generative fashion design simply by entering moderated data into parametric design algorithms using biomorphism as a knowledge base and using these to build an interface for people seeking new emotional communication for pleasure.

## Literature Review

Through a literature review, this study verified the applicability of the parametric design process in architecture to clothing sculpture and presented knowledge-based data to be used in the BCS interface and analytical items to be used in the case study, exploring parameters by examining the design approaches of biomorphic architectural form and parametric form generation methods.

### Clothing Sculpture as Architecture

As clothing has a 3D form and many parts that are connected based on the human body form, it is akin to a 3D architectural form, in which multiple elements are integrated into a structure. Moreover, the structural building method, details about connecting one part with another, and various physical properties used in architecture are similar to style, cutting and sewing methods, details, and fabrics used to make clothing. The 2006 *Skin + Bones: Parallel Practices in Fashion and Architecture* exhibition held at the Los Angeles Museum of Contemporary Art involved world-renowned architects and fashion designers. The exhibition displayed works in the two fields around the theme of new software materials and technology. Sculptor Zaha Hadid mentioned in an interview that both architecture and fashion exist for the well-being of users and that the basic principle of design is to explore emotional fragments in visual language. She claimed that both buildings and clothing are thus attractive 3D objects with functionality and esthetics, as they create style and pattern that reflects the paradigm of the times (Jury, 2008).

Building information modeling (BIM) is a technology that models not only design data for buildings but also all related information using a computer—from design to management and destruction of a building (Arayici, 2008). BIM is applied as a mediator variable in the parametric design process and helps solve problems in architectural design in which elaborate and diverse 3D forms must be developed. Kwon and Jun (2014), in a study of the design of framework materials for *hanoks* (traditional Korean houses), claimed that parametric design can use information on the architectural elements that form a building as various parameters to easily transform and adjust forms. In other words, they used the formative principles and geometric shapes of *hanoks* as a knowledge base and proposed a parametric design process based on how the materials are bonded as the mediator variable. They claimed that by altering the measurement of just one material in digital space, various other materials could also be altered easily and automatically, which enabled them to promptly assess the effect of modifying the material measurements. Based on this, they adapted the parametric design method used in architecture to the study of the BCS interface and used parameters in the knowledge-based modeling process as exploratory tools.

The advancement of new materials and technology, as well as 3D printing, has provided a completely new definition of tailoring. 3D modeling is becoming an innovative design process, as it uses scanning technology to design fashion items that perfectly fit the human body form. Farahi (2017) called for research on clothing made from flexible materials that produces harmony with the human body using geometry and proposed a study to develop materials that resemble human skin, based on anatomist Karl Langer’s idea that topological lines could be mapped onto the human body to represent the lines of skin tension. The scope of the application of research on BCS using the parametric design process with biomorphic inspiration as the knowledge base can be expanded in association with the development of 3D printing fashion design.

### Biomorphic Design

Biomorphic design, biophilic design, and biomimicry are all terms based on the concept of biomorphism. Biomorphism is a compound of “bio,” a complex term for life, lively phenomena, and biotics, and “morphism,” the collective form or composition of an organ or part (Alloway, 1975). Sculptor Jean Arp is referred to as the father of biomorphism (Greenbaum, 1994). Edward B. Henning, curator of the Cleveland Museum of Art, argued that Arp’s wood relief, *Forest*, influenced Joan Miro’s early experimental work in biomorphism, *The Hare* (Henning, 1979). Biomorphism is based on Henri Bergson’s philosophy of vitalism, which had a significant effect on French esthetics in the twentieth century. Bergson interpreted that the driving force for the evolution of “*élan original*” is “*élan vital*,” or dynamism and claimed that various images created by the movement of life are immaterial and comprised of the continuity of time that changes every hour. Intuition is perceived as shared intellect and instinct (Khandker, 2013; Manfred, 2016). Bergson’s philosophy influenced expressional characteristics of biomorphic art such as organic form, abstraction of vital movement, continuity of the object of expression, and automatism. Works by biomorphic artists followed. Arp referred to his sculptures as “concretion”; they expressed life in abstract, organic volumes of round shapes depicting the abundance and fullness of the growth and proliferation of nature (Rider, 2015). Miro identified a large tree, a blade of grass, an animal or snail, lizard, or small insect with life that has the same value as the entire universe. He freely modified this relationship through surrealist automatism and expressed abstract life forms that drift across the canvas (Adamowicz, 2012). Henry Moore detected the order of nature from primitive art filled with vitality and newly interpreted living organisms (Chryssovitsanou, 2013). Influenced by Miro and Arp, William Baziotes turned the growth of protozoa that drift in the ocean into abstract, wobbly lines and light expressed as vibration (Naves, 1995). Gaudi’s La Sagrada Familia is also considered a typical biomorphic building, as its pillars, which support the ceilings within, were inspired by the vitality and dynamics of trees. Gaudi designed this solid structure by imitating not only the external and natural form of trees but also their natural power (Joye, 2006).

Biophilic design that emerged with the popularization of biophilia in the 1980s and biomimicry, which emerged for sustainability, has inherited the attributes of biomorphic art that originated in modernism in the early twentieth century (Messenger, 2004). Edward O. Wilson, the progenitor of sociobiology, coined the term “biophilia” as a compound of “bio” and “philia” in 1979 to mean “living things have love of the natural environment.” Based on this meaning, he proposed biophilia as a formula whereby humans become one with living nature and achieve creative evolution as they are moved and healed (Joye, 2011).

Biomimicry refers to the biomimetics of the basic structure, principles, and mechanisms of living things and biomaterials in nature (Messenger, 2004). The term is derived from the Greek “*bio*” and “*mimicry*” and refers a design approach that mimics nature’s models, systems, and elements to solve complicated human problems. Janine Benyus, while not coining the term “biomimicry,” contributed to its generalization *via* her book *Biomimicry: Innovation Inspired by Nature*, which explains the early principles of biomimicry. She perceived nature as the object of the model, measure, and mentor and presented sustainability as the purpose of biomimicry (Symeonidou, 2019). Biomorphic design shares the concept of biomimicry when it uses natural motifs for the purposes of human welfare and healing—the fundamental concepts of biophilia. Hence, this study discusses biophilia along with the concept of biomorphic design. Cases of biomimicry include the Eiffel Tower, whose pattern and fractal form is inspired by high bone structure; the BionicMotionRobot (Festo, 2017), which mimics the movements of life; the high-speed railway in Japan, which reduces noise caused by aerodynamics inside tunnels by drawing inspiration from the silence of the kingfisher’s beak and owl’s feathers (Khaliq et al., 2014; Kennedy and Marting, 2016; Hayes et al., 2019); and Zimbabwe’s Eastgate Center, which reduces energy requirements by employing a ventilation system that imitates the structure of a termite nest (Ramzy, 2015). Thus, biomimicry—which mimics nature’s biological systems or structures—is becoming a solution in multiple fields, from everyday life to industry.

**Figure 1:**
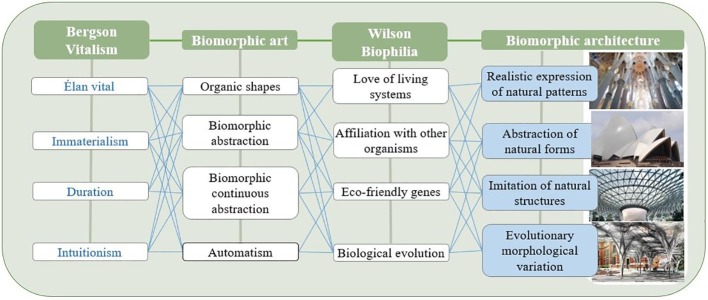
The types of the present biomorphic design approaches derived from Bergson’s theory of vitalism and characteristics of biomorphic art and biophilia.

Joye (2006) analyzed the attributes of biomorphic expressions in architecture as visual and structural sharing of nature’s models; imitation of natural patterns integrating biological patterns, textures, and sculptures; and emotional expressions of the evolution of trees or plants dominated by fractal geometry. He claimed that these attributes could become the grammar of form generation, producing creative and artistic outcomes. Fibonacci spirals and phyllotaxis patterns were earlier applied to the golden ratio and the Canon of Proportions in *The Vitruvian Man* and are still inspiring architects today (Symeonidou, 2019). Previous studies of biomorphic design combined with technology mentioned images such as birds’ nests, honeycomb, wings, twigs, radiolarians, onion cells, cabbage sections, human bones, spider webs, wavy wood, seashells, sponges, dripping water, shoals of fish, and *Sepia pharaonis* cuttlefish as motifs of biophilic emotions. These are applied to parametric methodology and are becoming sources of inspiration for modeling of furniture or buildings (Messenger, 2004; Vincent and Garcia, 2009; Mirkia et al., 2018; Symeonidou, 2019). Figure 1 illustrates types of contemporary biomorphic design approaches derived from Bergson’s theory of vitalism and characteristics of biomorphic art and biophilia.

### Parametric Design

Parametric design is a computer representation method that creates forms as design attributes and turns them into parameters as geometrical elements (Barrios Hernandez, 2006). In a parametric model, parameters can be altered to find new alternative solutions to a problem (Lotfabadi et al., 2016). Parametric design includes two modeling techniques. One is a more elaborate parametric modeling method that integrates a knowledge-based system, so that all elements are modified in terms of size and location through parameters of the relationship. This has the benefit of enabling the designer to predict the outcome of the parametric design. The other is a design method based on form generation, which programs designers’ ideas and related designs by entering them directly into a computer (Barrios Hernandez, 2006). The parameter refers to a certain factor or element that serves as a boundary or can be quantified. It is applied as a figure that can be quantified, but it may also include a functional standard such as light or structural resistance or a conceptualized esthetic standard used by designers to develop their own creative ideas. The parametric process is generated as algorithms *via* which a designer’s ideas materialize. The generated algorithms can create and modify various scenarios by adjusting the original parameters such as the shape’s location, scale, and angle depending on the concept; or the user may obtain output that satisfies their intentions through simulation (Abdullah and Kamara, 2013). The outcomes of parametric design may vary depending on the design procedures, design ideas, and tools or software used.

The process of clothing sculpture modeling involves parametric procedures related to the human body. Clothing is not only worn by a 3D human body but has various design components related to form, which makes it difficult to operate using a conventional digital design process alone. Therefore, there is a need for research on a parametric design process algorithm that can model curves and complicated forms. With information obtained from nature, the geometric principles and esthetics of the parametric design process form generation technique may lead to creative design outcomes in the BCS parametric design process, as the tool can bring comfort and pleasure to people today, whose everyday lives are linked to digital devices.

Parametric form generation is related to the generative principles of fractals, tessellation, and Voronoi. Fractal geometry was introduced in the 1970s when Benoit Mandelbrot declared that most biological forms have a fractal structure and was classified as a new geometric concept characterized by self-similarity, repeatability, and endless irregularity (Stamps, 2002; Vaughan and Ostwald, 2010; Rian and Asayama, 2016). In a fractal, shapes of different sizes are in a geometric form, but the location of the shapes or rotation of the forms is depicted in different states and magnifications (Stamps, 2002). The magnification of a fractal is not accurate, unlike in Euclidean geometry where magnifications are fixed by precise numbers, representing the complexity of the fractal pattern (Joye and Van Locke, 2007). As an example, Vaughan and Ostwald (2014) pointed out that the form of a tree from a distance is similar to the stem structure and leaf tissues of the same tree and claimed that a fractal structure is characterized by consistent complexity or irregularity of scales that intersect. Sedrez and Pereira (2012) stated that fractal geometry is depicted as rough, holey, or broken objects and is generated by a recurrent process in which the initiator and generator are repeated infinitely. Thus, a part is similar to or the same as the whole or has self-similarity that consistently follows the initial form in which everything is similar. In the generator, lines and faces, crooked lines, and non-linear forms can be used, and this generator is used in the algorithm to depict the final form. Even in the same algorithm, generators can be varied to represent different forms. In other words, the generative principle of the fractal algorithm is a feedback algorithm in which certain figures are entered, and the results are calculated through a fractal equation, which is then replaced according to the results (Rian, 2018). Accordingly, fractal algorithms are used in science, engineering, and medicine to explore and model non-linear and complicated shapes, as well as in creative concepts of art (Rian and Asayama, 2016).

The Voronoi diagram defined by mathematician Voronoi in the early twentieth century received attention as a very useful diagram that can explain phenomena and structures in nature. It is used in the form generation process of architecture, jewelry, and industrial design, as well as in various other fields such as computer engineering, meteorology, geology, topography, archeology, medical science, molecular chemistry, and ecology (Kim and Kim, 2006; Zheng et al., 2011; Fantini et al., 2016). The Voronoi diagram is an outcome of specific space division whereby, starting from a discontinuous point referred to as the “site,” all cells are comprised of points that are closer to the generation seed (Lautensack, 2008; Malinauskas, 2008; Phillips, 2014). All intersections that are not empty are collected to form a Voronoi diagram limited by the domain and used to model the space from a series of calculated points (Sainlot et al., 2017). With these characteristics, the Voronoi diagram is positioned as a generative mechanism of digital space combined with computer programming, explaining the focus on 3D space generation of Voronoi in the parametric design process of clothing sculpture. Clothing is comprised of a regular system following the curves of the human body that constantly evolves based on changes in the design components and is characterized by self-organization in which spontaneous patterns are formed. Through self-organization, a single pattern proliferates repeatedly, which seems to be an evolutionary process. 3D Voronoi that materializes through the reciprocal self-organization technique can be seen in natural phenomena that are easily observed, cell division, patterning in animals, and natural environments.

Tessellation is used to refer to faces that are filled horizontally when generated by the connection of Voronoi points. As the edges of the tessellation are used only to define the shape of each Voronoi cell, they can be used in the same sense as the Voronoi diagram. However, the current study considers Voronoi in terms of 3D form generation and tessellation in terms of 2D patterns. The attributes of each Voronoi cell generation point are defined as solid or empty, and related cells have the same attributes as the first generation point (Chow et al., 2007). These attributes are also related to the pattern generative principles of tessellation. The infrastructure of many patterns has three regular tessellations: triangles, squares, and regular hexagons. Squares, which are most easily applied, are popular (Sarhangi, 2012), but triangles are most commonly used, as they are effective in expressing simplicity and flexibility because the vertices are located on the same plane, unlike other polygons (Werghi, 2011). Tessellation, in which small, similar parts gather into an aggregation to form a huge whole, is prolific in the natural world and can be observed through an electron microscope; it thus serves to express the important grammar of parametric design, in which the forms of the natural world are computed.

## Research Method for the Biomorphic Clothing Sculpture Conceptual Parametric Design Process

**Figure 2:**
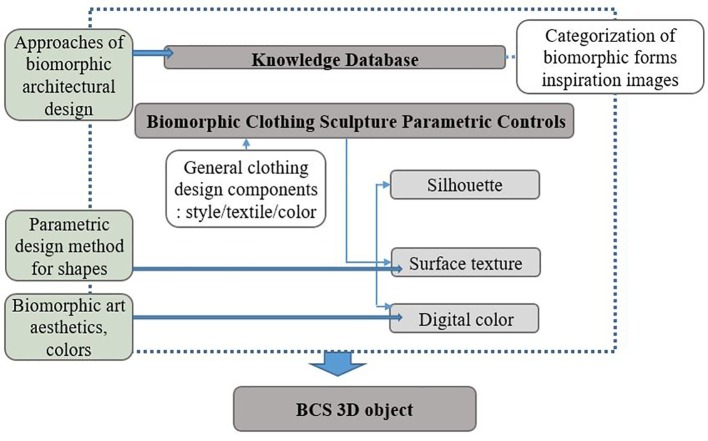
Conceptual parametric design process for the BCS.

This study aims to develop the conceptual interface applied by the categorization of biomorphic inspired images and 3D printed clothing design components as revealed through case studies as procedural parameters. In this study, parametric design methodologies and processes are used as a modeling method for the BCS interface. Based on the esthetics and colors of biomorphic art covered in the literature review, form generation approaches used in biomorphic architectural design, and parametric design process theories, this study proposes a conceptual parametric design process for the BCS interface as an emotional communication space. To prove the practicality of the conceptual interface, 126 biomorphic design cases and 124 3D printing clothes cases are collected and categorized into parameters. The representative examples of the case analysis parameters are illustrated as figures drawn or presented in the form of 3D modeling instances to demonstrate the results. Figure 2 shows the conceptual parametric design process for the BCS formed as a figure with reference to the parametric design process of architecture.

## Case Study

### Knowledge Database Case Study: Biomorphic Inspiration Images

**Figure 3:**
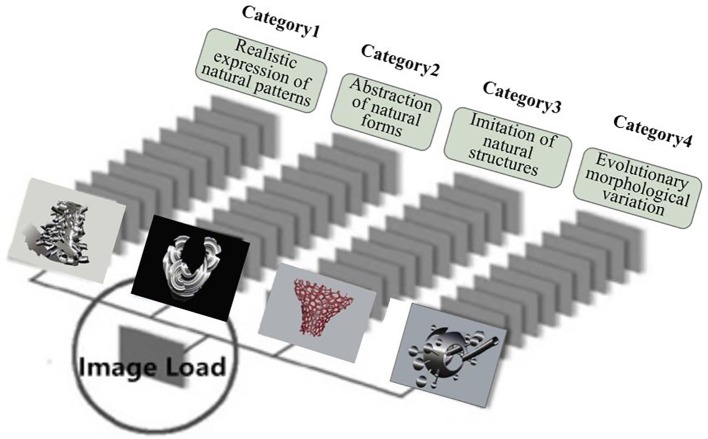
Categorization of biomorphic forms inspiration images.

As the knowledge database for the BCS interface, this study used biomorphic inspiration images from which generative design outcomes were predicted based on the findings of the literature review. The selected images were those created as 3D artistic forms or designs that referred to the biomorphic design form generation approach. Keywords such as “biomorphic shape,” “biomorphic form,” and “biomorphic structure” were used in the search, and one was selected only when similar forms were repeated for the same concept, ultimately resulting in the collection of a total of 126 cases. The cases were classified based on the form generation approach (Figure 1) to biomorphic architectural design summarized in the theoretical background and analyzed in categories such as “realistic expression of natural patterns,” “abstraction of natural forms,” “imitation of natural structures,” and “evolutionary morphological variation.” “Realistic expression of natural patterns” is a category that imitates nature and embodies animals and plants, reproducing them by instilling design opinions, rather than by merely imitating natural objects, thereby adapting the vitality of nature in the form of animals and plants and expressing it in similar natural form. “Abstraction of natural forms” is a method that expresses the mobility and naturalness of form represented by non-linear, free curves in the abstracted form of nature. Gentle curves and vivid curved surfaces are used to express visual changes and depth. “Imitation of natural structures” is a category of form represented by imitating the interaction and interconnectivity of nature, which takes the form of self-organization and is expressed similarly in cell structure. “Evolutionary morphological variation” is a structure similar to cell division and evolution that turns out to be a variation of a heterogeneous and unrealistic form. Figure 3 summarizes the major cases of biomorphic images categorized into these four types.

### Case Study of Parameters Moderating the Biomorphic Clothing Sculpture

A case study was conducted of 3D-printed clothing sculptures with BCS characteristics to explore parameters used in BCS modeling during the parametric design process of the BCS interface. The cases were those released as 3D-printed clothing. Keywords such as “3D printing fashion,” “3D printing designer,” “3D printing wearable fashion,” “3D biomorphic fashion,” “3D nature-inspired fashion,” and “3D biomimicry fashion” were used in the search, and a total of 124 cases were collected. The cases were analyzed based on the theory of biomorphic design expression characteristics and parametric design form generation principles. Three components of ordinary clothing were used as mediator variables, and finally, parameters were moderated to “silhouette,” “surface texture,” and digital color related to BCS composition. The results of the case study were applied as parameters in the BCS interface design, and the results are presented as photos with a focus on those that best represent the attributes of each mediator variable. When the results could not be explained by the photos collected, BCS modeling algorithms were established using a rhinoceros and a grasshopper, and exemplar photos are used to present the case.

#### Silhouette

**Figure 4:**
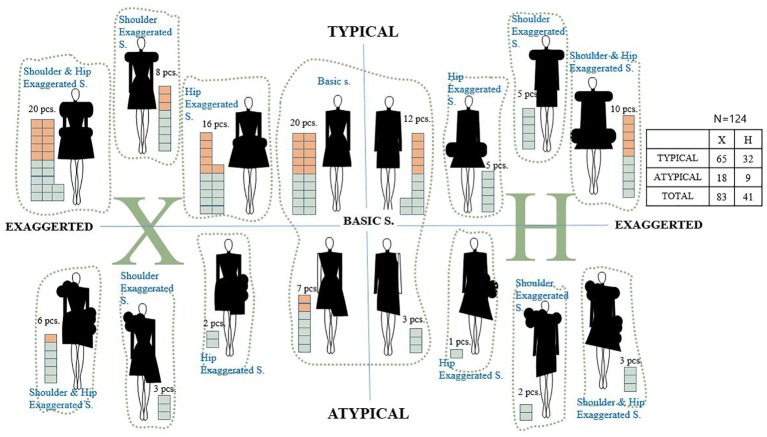
Categorization of BCS silhouettes through cases study.

BCS is a 3D structure with a *Z*-axis instead of a flat 2D plane, and its application to fashion design requires consideration of the shape of the human body form. Usually, a design is comprised of the structure of design principles that create the surface effect, and the principle of BCS is organically related to the human body and materialized complex, integrated esthetic order. Thus, of the internal lines and silhouette that are the formative components of general clothing, only the silhouette was used as a mediator variable. The general clothing silhouette was classified by shape based on letters of the alphabet such as H and X, or subdivided into I, X, A, and Y, as well as by shapes of objects such as an arrow, hourglass, tulip, tunic, balloon, cocoon, princess, empire, or mermaid. The BCS in the case study turned out to be a more exaggerated silhouette than that of general clothing, perhaps owing to the 3D printing material and texture. Therefore, this study divided the most fundamental silhouettes of H and X into symmetrical typical and asymmetrical atypical and categorized them into four types. As the result of a case analysis based on the above, they were again subdivided into basic silhouette (s.), “shoulder exaggerated s.,” “hip exaggerated s.,” and “shoulder, hip exaggerated s.,” with a focus on the horizontal width of the shoulders, waist, and hips. To achieve these distinctions, the user can make diverse and easy choices regarding procedures for form generation and processing in the interface. Accordingly, the user can choose from a total of 16 silhouette options. Figure 4 shows the categorization of BCS silhouette types; the most common case is 3D-printed clothing in the typical X silhouette.

#### Surface Texture

**Figure 5:**
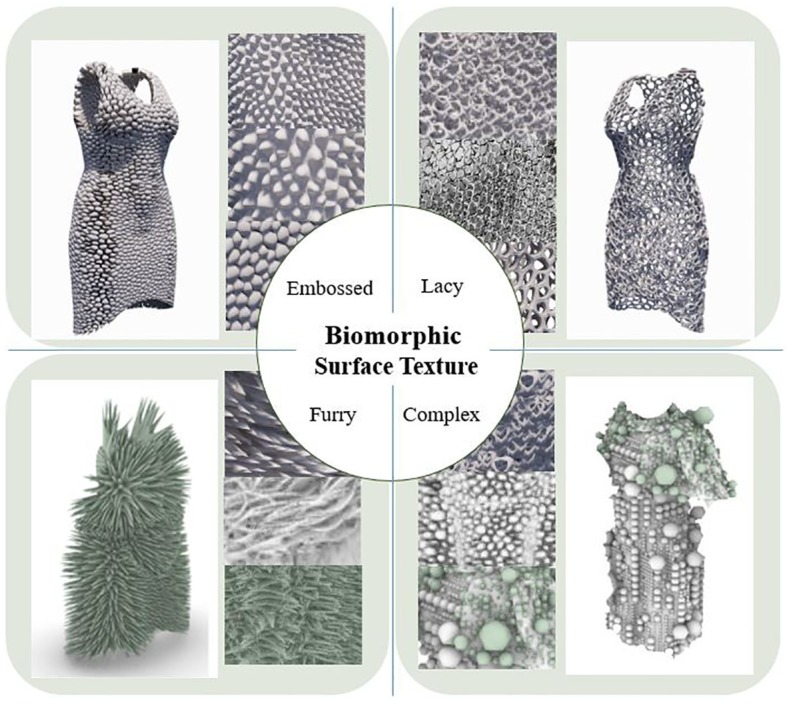
Categorization of BCS surface texture.

Materials of BCS applied to the parametric design methodology were classified with a focus on the surface texture that occurs by adjusting the measurements of pattern size or thickness in the parametric form modeling process. In other words, BCS surface textures that might vary depending on unevenness or density of pattern size were categorized as “embossed,” a type that shows differences in fabric surface thickened with embossing or pattern; “lacey,” which has a netted or laced surface”; “furry,” a texture of long fur like animal fur; or “complex,” in which multiple textures are mixed together. Figure 5 illustrates typical cases of 3D-printed clothing sculptures for each of the four surface texture categories. Photos are also presented of examples embodied by adjusting the parameters of the BCS form algorithms developed to explain the clear distinctions among textures.

#### Digital Color

**Figure 6:**
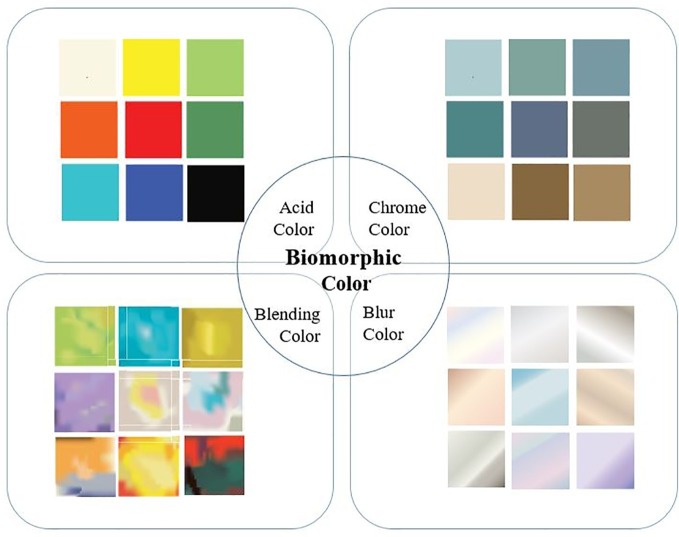
Categorization of BCS color.

Digital color directly perceives light through color and is physically and emotionally differentiated from general color perceived through the process of light absorption and reflection. Interface color is based on the attributes of light and may appear not only in collage images but also in multimedia or video clips; it is digital color that expresses emotional symbols for user communication. The interface has immaterial color characteristics that are non-fixed, amorphous, and non-constructive given a perception of continuous scenes or flow of time and space in which multiple faces are connected. Based on these color characteristics of the interface, this study used the color emotions and dominant colors that appear in artworks in the background of biomorphic art. YR and DK tones are used in biomorphic expressionist art to express artists’ subjective emotions and responses based on objects and events, rather than objective facts. BCS colors used in the interface are mainly digital colors classified into acid colors using medium brightness and high chroma with high attractiveness, which excludes achromatic colors; blending colors evolving from the phenomenon in which two or more colors are physically mixed; blur color that forms a gradation in which three color elements change according to irradiation or tones disappearing; and chrome color with metallic expressions added to produce achromatic glossy material or a metallic feel of silver and platinum. Figure 6 summarizes typical cases of 3D-printed works of each type as well as the four types of digital color extracted from the cases of biomorphic expressionist artwork.

## Conclusion: Proposal for the Biomorphic Clothing Sculpture Conceptual Interface

This paper presents a conceptual BCS interface that uses biomorphic design as a knowledge base using parametric design methodology from 3D form development of architecture. Rather than focusing on the technical aspects of 3D modeling, this study focused on developing an interface as a user-friendly playground with clothing sculpture modeling as the intermediary. A literature review and case study were conducted to gather knowledge-based data on interface and mediator variables for the parametric design process, and biomorphic design image categories were created using knowledge-based data that can predict biomorphic forms. The study also categorized silhouette, surface texture, and digital color as parameters required for building 3D BCS forms and determined the subordinate attributes of each mediator variable.

**Figure 7:**
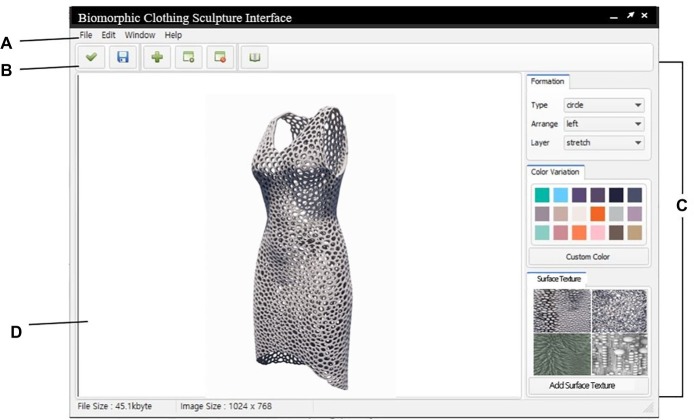
Conceptual task pad of the BCS interface.

**Figure 8:**
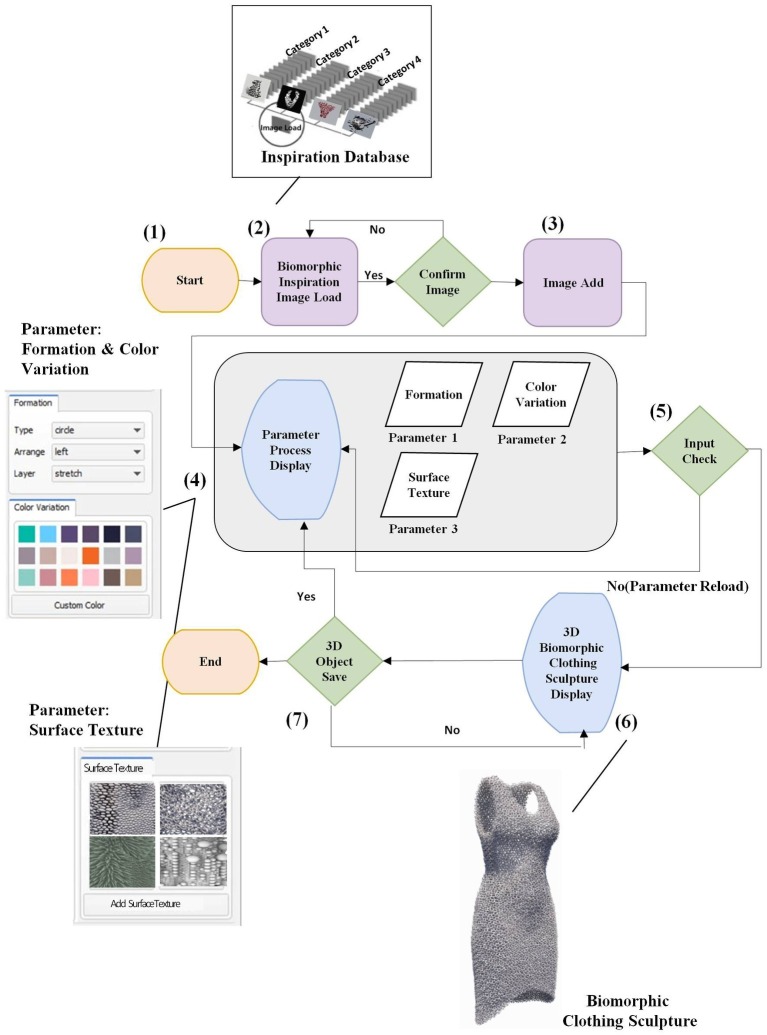
The procedures for using the BCS 3D modeling interface.

Figure 7 illustrates the conceptual task pad of the BCS interface, which is divided into four parts: A, B, C, and D. There are five parts including the saved biomorphic inspiration image database. The images supported by the biomorphic inspiration image database category are saved in the database and can be loaded for use. The biomorphic inspiration image database is categorized, and thus, it is easy to save and process images. Data are saved in blob (binary) format. A collection of research images is provided, to which users can add more. A is comprised of four categories: File, Edit, Window, and Help. In File, the user can load an image file or save a created image. The user can perform basic tasks with the loaded image such as selecting, enlarging, and reducing it in the toolbox B or Edit tab. Specific image modification is carried out in C. C is a toolbox comprised of form generation mediator variables. The BCS interface changes the names of the parameters “silhouette” and “digital color,” derived from the case study, to “formation” and “variation color,” to make it easier for users to access them. In formation, detailed form characteristics of BCS for adjusting the shape are given as numbers. Multiple parameters can be selected, and there is no separate order of application. Moreover, detailed settings are possible for each mediator variable, and the values must be entered as numbers or ratios. Surface texture is determined through four techniques extracted from the case study, and the selected technique creates expressive technique algorithms through mathematical operations that adjust thickness or pattern size, and form generation methods of fractals, tessellation, and Voronoi, thereby altering the surface. In terms of color variation, types of colors extracted from the case study were used as mediator variables. Colors other than the proposed types can be added by users. Moderated images as parameters are generated as 3D objects according to parameters such as image, form, color, and technique, selected on the D monitor. Figure 8 demonstrates the procedures for using the BCS 3D modeling interface.

This study is significant in that it used biomorphic design with various concepts and ambiguous emotions as knowledge-based data to predict the results of the parametric process as well as the forms of clothing sculpture interworked with fashion that changes with time as parameters through a case study. The results of this study will be provided as a user-friendly interface that is accessible to users simply by clicking on the option tool comprised of mediator variable items and will be thoroughly reviewed *via* future case studies.

In addition, this study was focused on the development of the conceptual interfaces; thus, the paper does not include descriptions of computational terms for modeling cases. Instead, we are preparing a further paper focusing on the modeling case of clothing sculpture, which will include a description of the modeling process in computational terms.

## Data Availability Statement

The datasets generated for this study are available on request to the corresponding author.

## Author Contributions

All authors listed have made a substantial, direct and intellectual contribution to the work, and approved it for publication.

### Conflict of Interest

The authors declare that the research was conducted in the absence of any commercial or financial relationships that could be construed as a potential conflict of interest.

## References

[ref1] Abdullah H. K. Kamara J. M. (2013). Parametric design procedures: a new approach to generative-form in the conceptual design phase. ASCE 334–343. 10.1061/9780784412909.032

[ref2] Adamowicz E. (2012). Joan Miró: the assassination of painting? J. Iber. Lat. Am. Stud. 18, 1–15. 10.1080/14701847.2012.716642

[ref3] Alloway L. (1975). Topic in American art since 1945. New York: W. W. Norton & Company Inc.

[ref4] Arayici Y. (2008). Towards building information modelling for existing structures. Struct. Surv. 26, 210–222. 10.1108/02630800810887108

[ref6] Barrios Hernandez C. (2006). Thinking parametric design: introducing parametric Gaudi. Des. Stud. 27, 309–324. 10.1016/j.destud.2005.11.006

[ref7] Chow H. Tan S. Sze W. (2007). Layered modeling of porous structures with Voronoi diagrams. Comput.-Aided Des. Appl. 4, 321–330. 10.1080/16864360.2007.10738552

[ref8] Chryssovitsanou V. (2013). Henry Moore et l’art cycladique. Les Nouvelles De L’Archéologie 134, 5–11. 10.4000/nda.2219

[ref9] Fantini M. Curto M. De Crescenzio F. (2016). A method to design biomimetic scaffolds for bone tissue engineering based on Voronoi lattices. Virtual Phys. Prototyping 11, 77–90. 10.1080/17452759.2016.1172301

[ref10] Farahi B. (2017). Material Behaviours in 3D-printed fashion items. Archit. Des. 87, 84–91. 10.1002/ad.2242

[ref11] Festo (2017). BionicMotionRobot. Available at: https://www.festo.com/group/en/cms/12747.htm (Accessed October 13, 2019).

[ref12] Greenbaum T. (1994). Bizarre bijoux: surrealism in jewelry. J. Decor. Propaganda Arts 20, 197–207.

[ref13] Hayes S. Desha C. Burke M. Gibbs M. Chester M. (2019). Leveraging socio-ecological resilience theory to build climate resilience in transport infrastructure. Transp. Rev. 39, 677–699. 10.1080/01441647.2019.1612480

[ref14] Henning E. (1979). A painting by Joan Miró. Bull. Cleveland Mus. Art 66, 235–240.

[ref15] Joye Y. (2006). Cognitive and evolutionary speculations for biomorphic architecture. Leonardo 39, 145–152. 10.1162/leon.2006.39.2.145

[ref16] Joye Y. (2011). Biophilic design aesthetics in art and design education. J. Aesthetic Educ. 45, 17–35. 10.5406/jaesteduc.45.2.0017

[ref17] Joye Y. Van Locke P. (2007). Motivating biomorphic constructions based on complex systems science. Syst. Res. Behav. Sci. 24, 103–114. 10.1002/sres.742

[ref18] Jury L. (2008). How architects work… after a fashion. Available at: https://www.standard.co.uk/news/how-architects-work-after-a-fashion-6624005.html (Accessed October 13, 2019).

[ref19] Kennedy E. Marting T. (2016). Biomimicry: streamlining the front end of innovation for environmentally sustainable products: biomimicry can be a powerful design tool to support sustainability-driven product development in the front end of innovation. Res. Technol. Manag. 59, 40–48. 10.1080/08956308.2016.1185342

[ref20] Khaliq A. Di Rocco A. Saffiotti M. (2014). Stigmergic algorithms for multiple minimalistic robots on an RFID floor. Swarm Intell. 8, 199–225. 10.1007/s11721-014-0096-0

[ref21] Khandker W. (2013). The idea of will and organic evolution in Bergson’s philosophy of life. Cont. Philos. Rev. 46, 57–74. 10.1007/s11007-013-9248-y

[ref22] Kim D.-G. Kim D.-S. (2006). Region-expansion for the Voronoi diagram of 3D spheres. Comput. Aided Des. 38, 417–430. 10.1016/j.cad.2005.11.007

[ref23] Kwon S. Jun H. (2014). A study on the representation of knowledge and use of Han- ok components based on parametric design. J. Archit. Inst. Korea Plann. Des. 30, 101–110. 10.5659/JAIK_PD.2014.30.7.101

[ref24] Lautensack C. (2008). Fitting three-dimensional Laguerre tessellations to foam structures. J. Appl. Stat. 35, 985–995. 10.1080/02664760802188112

[ref25] Lotfabadi P. Alibaba H. Arfaei A. (2016). Sustainability; as a combination of parametric patterns and bionic strategies. Renew. Sust. Energ. Rev. 57, 1337–1346. 10.1016/j.rser.2015.12.210

[ref26] Malinauskas K. (2008). Dynamic construction of abstract Voronoi diagrams. J. Math. Sci. 154, 214–222. 10.1007/s10958-008-9160-x

[ref27] Manfred M. (2016). Essay in honor of Robert Motherwell’s centenary: “temporalized form”: mediating romanticism and American expressionism—Robert Motherwell, Henri Bergson, and the ontological origins of abstraction around 18001. J. Aesthetics Cult. 8, 1–22. 10.3402/jac.v8.29952

[ref28] Messenger C. (2004). "their small-toothed interlock"1: Biomorphism and mystical quest in the visual art of P.K. page and John Vanderpant. J. Can. Stud. 38, 76–96. 10.3138/jcs.38.1.76

[ref29] Mirkia H. Nelson M. Assadi A. H. Abercrombie H. Nussbaumer L. Thorleifsdottir K. (2018). The impact of biomorphic design on the memorability of interior environments. proQuest dissertations and theses.

[ref30] Naves M. (1995). Ambiguous Baziotes. New Criterion 13, 38–41.

[ref31] Öhman A. Mineka S. (2001). Fears, phobias, and preparedness: toward an evolved module of fear and fear learning. Psychol. Rev. 108, 483–522. 10.1037/0033-295X.108.3.483, PMID: 11488376

[ref32] Phillips D. (2014). Tessellation. Wiley Interdiscip. Rev.: Comput. Stat. 6, 202–209. 10.1002/wics.1298

[ref33] Ramzy N. (2015). Sustainable spaces with psychological values: historical architecture as reference book for biomimetic models with biophilic qualities. Archnet-IJAR 9, 248–267. 10.26687/archnet-ijar.v9i2.464

[ref34] Rian I. (2018). Fractal-based computational modeling and shape transition of a hyperbolic Paraboloid Shell structure. Nexus Network J. 20, 437–458. 10.1007/s00004-018-0394-8

[ref35] Rian I. Asayama S. (2016). Computational design of a nature-inspired architectural structure using the concepts of self-similar and random fractals. Autom. Constr. 66, 43–58. 10.1016/j.autcon.2016.03.010

[ref36] Rider A. (2015). The concreteness of concrete art. Parallax 21, 340–352. 10.1080/13534645.2015.1058887

[ref37] Sainlot M. Nivoliers V. Attali D. (2017). Restricting Voronoi diagrams to meshes using corner validation. Comput. Graphics Forum 36, 81–91. 10.1111/cgf.13247

[ref38] Sarhangi R. (2012). Interlocking star polygons in Persian architecture: the special case of the decagram in mosaic designs. Nexus Network J. 14, 345–372. 10.1007/s00004-012-0117-5

[ref39] Sedrez M. Pereira R. (2012). Fractal shape. Nexus Network J. 14, 97–107. 10.1007/s00004-011-0099-8

[ref40] Söderlund J. Newman P. (2017). Improving mental health in prisons through biophilic design. Prison J. 97, 750–772. 10.1177/0032885517734516

[ref41] Stamps A. E. (2002). Fractals, skylines, nature and beauty. Landsc. Urban Plan. 60, 163–184. 10.1016/S0169-2046(02)00054-3

[ref42] Symeonidou I. (2019). Epidermis: algorithmic design based on biomimetic morphology. Nexus Network J. 21, 161–174. 10.1007/s00004-018-0412-x

[ref43] Ulrich R. (1984). View through a window may influence recovery from surgery. Science 224, 420–421. 10.1126/science.6143402, PMID: 6143402

[ref44] Vaughan J. Ostwald M. (2010). Using fractal analysis to compare the characteristic complexity of nature and architecture: re-examining the evidence. Archit. Sci. Rev. 53, 323–332. 10.3763/asre.2010.0024

[ref45] Vaughan J. Ostwald M. (2014). Measuring the significance of façade transparency in Australian regionalist architecture: a computational analysis of 10 designs by Glenn Murcutt. Archit. Sci. Rev. 57, 249–259. 10.1080/00038628.2014.940273

[ref46] Vincent J. Garcia M. (2009). Biomimetic patterns in architectural design. Archit. Des. 79, 74–81. 10.1002/ad.982

[ref47] Werghi N. (2011). Assessing the regularity of 3D triangular mesh tessellation using a topological structured pattern. Comput.-Aided Des. Appl. 8, 633–648. 10.3722/cadaps.2011.633-648

[ref48] Zheng W. T. Xu W. Y. Yan D. X. Ji H. (2011). Communications in computer and information. Science 228, 62–69. 10.1007/978-3-642-23223-7_8

